# Obesity or Overweight Is Associated with Worse Pathological Response to Neoadjuvant Chemotherapy among Chinese Women with Breast Cancer

**DOI:** 10.1371/journal.pone.0041380

**Published:** 2012-07-25

**Authors:** Sheng Chen, Can-Ming Chen, Ying Zhou, Ruo-Ji Zhou, Ke-Da Yu, Zhi-Ming Shao

**Affiliations:** 1 Department of Breast Surgery, Fudan University Shanghai Cancer Center/Cancer Institute, Shanghai, P.R. China; 2 Department of Oncology, Shanghai Medical College, Fudan University, Shanghai, P.R. China; 3 Department of Pathology, Fudan University Shanghai Cancer Center/Cancer Institute, Shanghai, P.R. China; 4 Institutes of Biomedical Science, Fudan University, Shanghai, P.R. China; Virginia Commonwealth University School of Medicine, United States of America

## Abstract

**Background:**

To evaluate the relationship between body mass index (BMI) and response to neoadjuvant chemotherapy (NCT) for breast cancer among Chinese women.

**Patients and Methods:**

A total of 307 eligible patients were assigned to receive four cycles of paclitaxel and carboplatin before standard surgery for breast cancer from 2007 to 2011 at Shanghai Cancer Hospital. The patients were categorized as obese, overweight, normal weight, or underweight based on BMI according to World Health Organization (WHO) criteria. Pathological complete response (pCR) was defined as no invasive cancer in the breast or axillary tissue. A logistic regression and the Chi-squared test were used for detecting the predictors of pCR and determining the relationship between BMI category and pCR rate in the subgroup analysis with respect to other variables.

**Results:**

Categorical BMI, estrogen receptor (ER), and progesterone receptor (PR) status were independent predictors of pCR according to the multivariate analysis. Patients with BMI≥25 were less likely to achieve a pCR to NCT compared with patients with BMI<25 (Odds ratio: 0.454, p = 0.033, multivariate analysis). In the subgroup analysis, the predictive value of BMI for pCR to NCT was significantly shown in post-menopausal patients (p = 0.004) and hormonal receptor status-negative patients (p = 0.038). The incidence of treatment-induced toxicity was similar among the different BMI categories.

**Conclusion:**

Higher BMI was associated with worse pCR to NCT. Further approaches to investigating the mechanism of this influence of BMI on treatment response and a more appropriate schedule for calculating NCT dose for high-BMI-patients should be considered.

## Introduction

The measurement of body mass index (BMI), as a standard method to detect obese or overweight people, has been widely accepted to have an effect on the development and prognosis of breast cancer. High BMI has been associated with a higher risk of breast cancer in multiple studies [1], [2]. Obesity is also associated with advanced disease at diagnosis and with a poor prognosis in both pre-menopausal and post-menopausal women with breast cancer [3], [4], [5], [6]. The underlying mechanism of these effects remains unknown. Most investigators believe that higher BMI is related to higher circulating concentrations of sex hormones, insulin and insulin-like growth factor, which lead to a distortion of the normal balance between cell differentiation and apoptosis and the progression and proliferation of breast cancer cells [7], [8].

Some reports indicate that obese women with cancer would have a relatively poor response to treatment [9]; however, whether BMI affects chemotherapy sensitivity in breast cancer is still unclear. Neoadjuvant chemotherapy (NCT), which provides an opportunity to gain early information about the response to chemotherapeutic drugs, would most likely serve as the best model for a better understanding of this issue. A large study of 1169 breast cancer patients treated with NCT has demonstrated that BMI is associated with the probability of complete pathological response (pCR) [10]. Overweight patients and the combination of overweight and obese patients were significantly less likely to have a pCR. That is the first study to demonstrate the important role of BMI in predicting NCT response in breast cancer.

**Table 1:** Patient characteristics according to BMI.

Characteristic	Number of patients (%)			P value	
	Underweight/Normal	Overweight/Obesity	All		
Age				NS	
Median (Range)	51 (28–70)	53 (26–71)	52 (26–71)		
Menopausal status				0.027	
Pre	101 (54.0%)	49 (40.8%)	150 (48.9%)		
Post	86 (46.0%)	71 (59.2%)	157 (51.1%)		
Tumor stage				NS	
T2	73 (39.0%)	34 (28.3%)	107 (34.9%)		
T3	82 (43.9%)	56 (46.7%)	138 (45.0%)		
T4	32 (17.1%)	30 (25.0%)	62 (20.1%)		
Node status				NS	
−	52 (27.8%)	38 (31.7%)	90 (29.3%)		
+	135 (72.2%)	82 (68.3%)	217 (70.7%)		
Cancer stage				NS	
II	87 (46.5%)	50 (41.7%)	137 (44.6%)		
III	100 (53.5%)	70 (58.3%)	170 (55.4%)		
Histology				NS	
Invasive ductal carcinoma	124 (66.3%)	83 (69.2%)	207 (67.4%)		
Invasive (mixed) carcinoma	56 (29.9%)	35 (29.2%)	91 (29.6%)		
Others	7 (3.7%)	2 (1.7%)	9 (2.9%)		
ER status				NS	
−	67 (35.8%)	35 (29.2%)	102 (33.2%)		
+	120 (64.2%)	85 (70.8%)	205 (66.8%)		
PR status				NS	
−	62 (33.2%)	29 (24.2%)	91 (29.6%)		
+	125 (66.8%)	91 (75.8%)	216 (70.4%)		
HER-2 status				NS	
−	136 (72.7%)	94 (78.3%)	230 (74.9%)		
+	51 (27.3%)	26 (21.7%)	77 (25.1%)		

Abbreviations: BMI, body mass index; NS, no significance; ER, estrogen receptor; PR, progesterone receptor; HER-2, human epidermal receptor.

In China, there are less obese people (BMI≥30 according to W.H.O. criteria) compared with western countries. In recent years, with an increasing population of overweight breast cancer patients, this risk factor is playing an increasingly important and indispensable role in breast cancer treatment. The aim of our study is to detect the relationship between BMI and the pathological response to NCT and to determine whether this relationship varies among different subgroups of Chinese women with breast cancer.

## Materials and Methods

### Study Population

Between May, 2007 and August, 2011, 307 women with large operable (primary invasive tumor >3 cm and N0-1) or local advanced breast cancer, as confirmed by core needle biopsy, without prior treatment were selected for neoadjuvant chemotherapy (NCT) with weekly PC (paclitaxel plus carboplatin) followed by surgical resections at Shanghai Cancer Hospital. We retrospectively collected the complete data including treatment history, clinical examination, imaging examination (bilateral mammography, breast ultrasound and MRI), and pathological assessment. The inclusion and exclusion criteria for patients needing NCT was from one phase II trial to evaluate the activity and safety of weekly PC regimen as neoadjuvant treatment in women with locally advanced breast cancer or large operable disease [11]. Blood chemistry, bone scan, chest X-ray and abdominal ultrasound examination were performed to exclude metastatic disease before the initiation of the primary treatment. All patients’ node status was assessed through fine needle aspiration (FNA) of palpable lymph nodes before NCT. Bilateral breast cancer, male breast cancer and inflammatory breast cancer were not included in this study. Our study was approved by the independent ethical committee/institutional review board of Fudan University Shanghai Cancer Center (Shanghai Cancer Center Ethical Committee). All patients gave their written informed consent before inclusion in this study.

**Figure 1:**
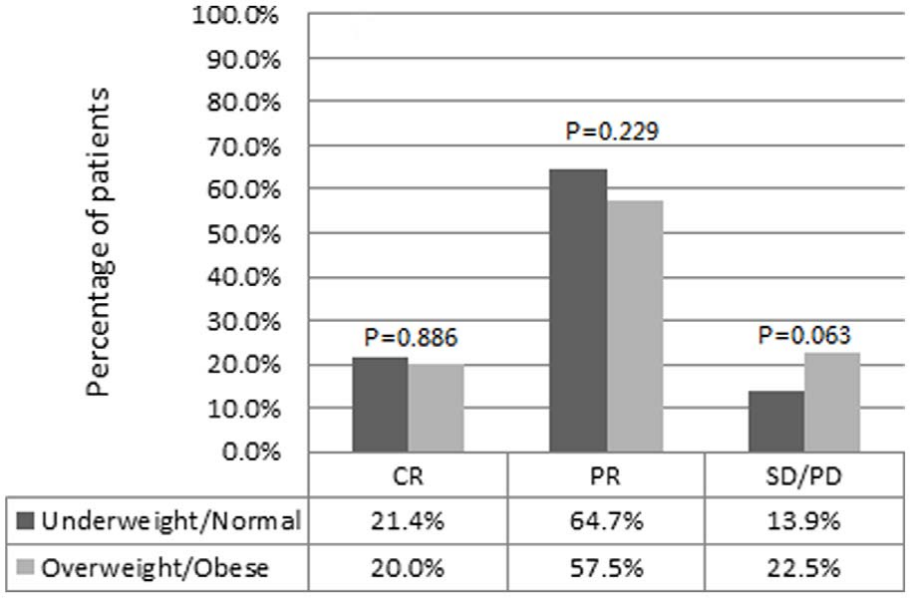
Clinical response to neoadjuvant chemotherapy and BMI category. CR, complete remission; PR, partial remission; SD, stable disease; PD, progression disease.

Patients’ BMI were calculated at enrollment as weight (kg) divided by height (m^2^), and the patients were separated into three groups: normal/underweight (BMI≤25 kg/m^2^), overweight (BMI between 25 and 30 kg/m^2^) and obese (BMI≥30 kg/m^2^) [12].

**Table 2:** Univariate and multivariate logistic regression models predicting pathological complete response after neoadjuvant chemotherapy.

Characteristics	Univariate model			Multivariate model		
	OR	95% CI	P	OR	95% CI	P
BMI						
Continuous	0.938	0.858–1.026	0.160	**–**	**–**	**–**
Categorical						
Normal/Underweight	1.000			1.000		
Overweight/Obesity	0.446	0.228–0.876	0.019	0.454	0.220–0.939	0.033
Age						
Continuous	0.982	0.952–1.013	0.252	0.997	0.948–1.048	0.886
Menopausal status						
Pre	1.000			1.000		
Post	0.626	0.344–1.139	0.125	0.628	0.236–1.672	0.352
Tumor stage						
T2	1.000			1.000		
T3	0.668	0.343–1.301	0.236	1.015	0.472–2.180	0.970
T4	0.843	0.378–1.881	0.677	1.111	0.453–2.724	0.819
Node status						
−	1.000			1.000		
+	0.871	0.460–1.646	0.670	0.819	0.400–1.680	0.587
Histology						
Invasive ductal carcinoma	1.000			1.000		
Invasive (mixed) carcinoma	0.809	0.414–1.579	0.534	0.902	0.433–1.876	0.782
Others	0.556	0.068–4.578	0.585	0.756	0.078–7.334	0.809
ER						
−	1.000			1.000		
+	0.211	0.113–0.394	<0.001	0.386	0.169–0.882	0.024
PR						
−	1.000			1.000		
+	0.185	0.099–0.347	<0.001	0.354	0.160–0.782	0.010
HER-2						
−	1.000			1.000		
+	1.869	0.992–3.522	0.053	1.091	0.529–2.253	0.813

Abbreviations: OR, odds ratio; BMI, body mass index; ER, estrogen receptor; PR, progesterone receptor; HER-2, human epidermal receptor.

### Treatment and Toxicity

All patients underwent four cycles of NCT with paclitaxel (80 mg/m^2^) and carboplatin (AUC 2 mg*min/ml) on day 1, 8, and 15 of a 28-day cycle. No other anti-cancer treatments, including chemotherapy, radiation therapy or endocrine therapy before surgery, were permitted. After completion of the NCT, all patients underwent mastectomy or breast conserving therapy (BCT) with sentinel lymph node biopsy (SLNB) or axillary lymph node dissection (ALND) within four weeks (if a patient was SLNB+, ALND was performed).

**Figure 2:**
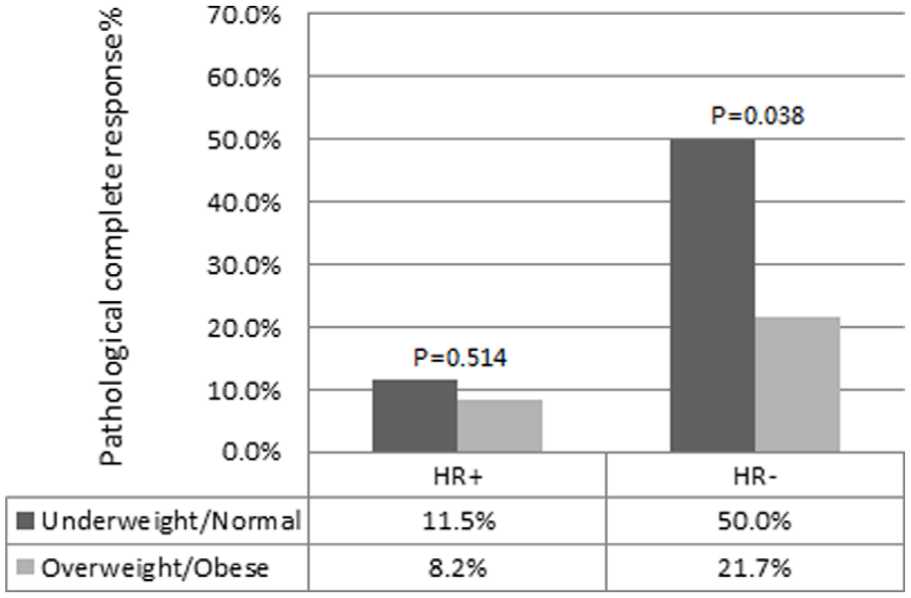
Probability of pathological complete response according to hormonal receptor status and BMI category. HR, hormonal receptor; HR+, ER+ or PR+; HR−, ER− and PR−.

The clinical response to neoadjuvant chemotherapy was evaluated based on MRI and according to response evaluation criteria in solid tumors (RECIST) 1.1 [13]. Clinical complete response (cCR) was defined as no clinical evidence of tumor in the breast and lymph nodes. Partial response (cPR) was defined as a greater than 30% reduction in the greatest tumor diameter. A reduction of less than 30% or an increase of up to 20% in the greatest tumor diameter was regarded as stable disease (cSD), whereas an increase of more than 20% in the greatest tumor diameter or the appearance of new disease was regarded as progressive disease (cPD).

**Figure 3:**
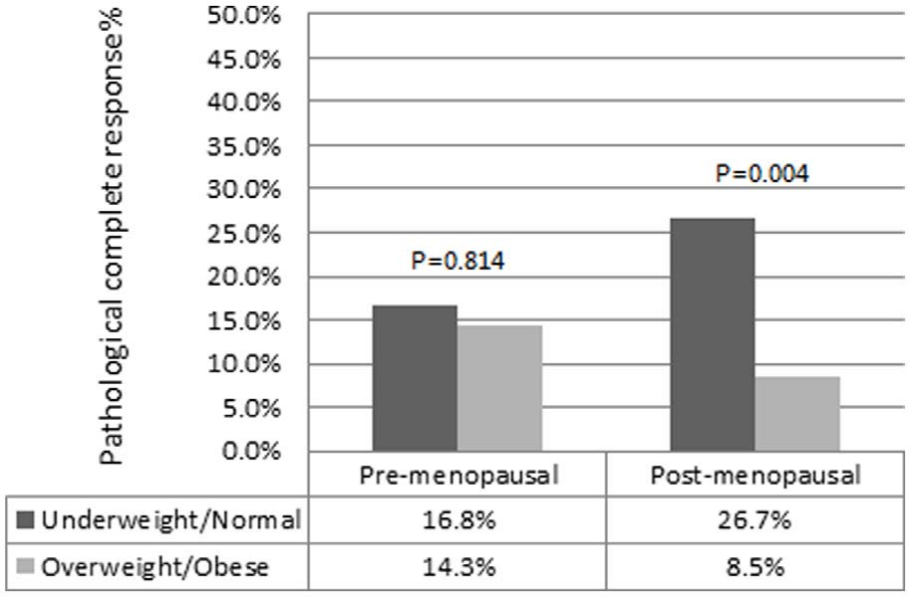
Probability of pathological complete response according to menopausal status and BMI category.

Toxicity was evaluated at the end of every NCT cycle and was recorded according to the NCI-CTC version 3.0.

### Pathology and Immunohistochemistry

All pathological evaluations were performed at Shanghai Cancer Hospital. The original histological determinations were obtained through CNB before enrollment. A pCR after NCT was defined as the absence of invasive carcinoma in both the breast tissue and lymph nodes of the resected specimen. Residual ductal carcinoma in situ (DCIS) was included in the pCR group.

Immunohistochemistry (IHC) analysis was performed on formalin-fixed, paraffin-embedded tissue sections using standard procedures for breast tumor specimens from CNB to evaluate the expression of estrogen receptor (ER), progesterone receptor (PR) and human epidermal growth factor receptor-2 (HER-2) prior to NCT. The cut-off value for ER positivity and PR positivity was 1% positive tumor cells with nuclear staining. HER-2 was evaluated as 0, 1+, 2+ or 3+ using circumferential membrane-bound staining, and positivity (HER-2+) was considered as 3+ using IHC or positive on FISH, whereas cases with 0 to 1+ or 2+ without FISH detection were regarded as negative (HER-2−). The following antibodies were used for IHC: ER (M7047, clone 1D5, Dako, Produktionsvej, Glostrup, Denmark), PR (M3569, clone PR 636, Dako), and HER-2 (A0485, polyclonal rabbit antibody, Dako).

### Statistical Analysis

The Chi-squared test was used to evaluate the relationship between patient characteristics and BMI category and to compare groups based on BMI. The Fisher exact test was performed when necessary. A multivariate logistic regression model for predicting pCR was used considering both categorical (BMI, menopausal status, tumor size, lymph node status, ER status, PR status, and HER-2 status) and continuous variables (age) evaluated at diagnosis. All statistical tests were two sided, and P values less than 0.05 were considered significant. All analyses were performed with SPSS (version 13.0, SPSS Company, Chicago, IL).

## Results

### Patient Characteristics and BMI Category

The median BMI of all patients before NCT was 23 (range: 17–33). Overall, 60.9% of the patients were classified as underweight or normal status based on BMI (n = 1 in underweight group and n = 186 in normal weight group); 33.6% of patients were classified as overweight (n = 103), whereas 5.5% of patients were classified as obese (n = 17). Because of the small number of patients in the obese category, obese and overweight patients were grouped together. **Table 1** shows the patient characteristics according to BMI category. Patient menopausal status was significantly associated with BMI category (p = 0.027). A higher percentage of overweight/obese patients was observed in post-menopausal women. However, age, tumor stage, node status, cancer stage, tumor histology, ER status, PR status and HER-2 status were not significantly correlated with BMI.

### Clinical Response and BMI Category

**** **Figure 1** shows the relationship between the clinical response to NCT and BMI categories. The overall clinical response rate was 82.7% with a cCR rate of 20.8%. In the underweight/normal category, a total of 40 (21.4%) patients experienced cCR, 121 patients (64.7%) experienced cPR, and 26 (13.9%) experienced cSD or PD. Furthermore, in the overweight/obese category, the rates of cCR, cPR and cSD/PD were 20.0%, 57.5% and 22.5%, respectively. There was a trend of a higher proportion of non-response patients (SD or PD) in the overweight/obese category compared with the underweight/normal category, but this difference was not statistically significant (p = 0.063).

### Pathological Complete Response and BMI Category

Among the 307 patients, the pCR rate was 17.3% (53/307). **Table 2** shows the univariate and multivariate analysis of the pCR predictors using a logistic regression model. In the univariate model, there was no significant relationship between pCR and BMI as a continuous variable (p = 0.160). However, BMI was significantly associated with pCR when considering it as a categorical variable by combining the overweight and obese categories (p = 0.019). Patients with a BMI≥25 (obese and overweight) were less likely to achieve a pCR to NCT compared with patients with a BMI<25 (normal and underweight). In the multivariate model, BMI (categorical), ER and PR status were independent predictors of pCR, with ORs of 0.454, 0.386, and 0.354, respectively. While considering BMI and hormonal receptor (HR) status together, underweight/normal women were more likely to have pCR compared with overweight/obese women for both HR+(either ER+ or PR+) and HR-(ER- and PR-) tumors (**figure 2**). However, the predictive value of BMI was significant in HR- patients (p = 0.038).

**Table 3:** Toxicity according to BMI category.

Toxicity	Number of patients (%)		
	Underweight/NormalN = 187 (100%)	Overweight/ObeseN = 120 (100%)	AllN = 307 (100%)
Anemia			
Grade 1–2	130 (69.5%)	78 (65.0%)	208 (67.8%)
Grade 3–4	4 (2.1%)	1 (0.8%)	5 (1.6%)
Thrombocytopenia			
Grade 1–2	57 (30.5%)	37 (30.8%)	94 (30.6%)
Grade 3–4	1 (0.5%)	0 (0.0%)	1 (0.3%)
Neutropenia			
Grade 1–2	99 (52.9%)	50 (41.7%)	166 (48.5%)
Grade 3–4	69 (36.9%)	34 (28.3%)	103 (33.6%)
Liver			
Grade 1–2	27 (14.4%)	16 (13.3%)	43 (14.0%)
Grade 3–4	2 (1.1%)	3 (2.5%)	5 (1.6%)
Neurotoxicity			
Grade 1–2	90 (48.1%)	51 (42.5%)	141 (45.9%)
Grade 3–4	0 (0.0%)	1 (0.8%)	1 (0.3%)
Nausea or vomiting			
Grade 1–2	169 (90.3%)	111 (92.5%)	280 (91.2%)
Grade 3–4	0 (0.0%)	0 (0.0%)	0 (0.0%)

In addition, BMI had exhibited different predictive values in pCR with respect to menopausal status (**figure 3**). It was significantly correlated with pCR in post-menopausal women (26.7% in underweight/normal category *vs.* 8.5% in overweight/obese category, p = 0.004). However, in pre-menopausal women, there was no significant difference in pCR with respect to BMI category (16.8% in the underweight/normal category *vs.* 14.3% in the overweight/obese category, p = 0.814).

### Toxicity

**Table 3** shows the main treatment-related toxicity observed in the patients. No episodes of death, symptomatic cardiac adverse events or life-threatening events were recorded. The incidences of hematologic toxicity, liver toxicity, neurotoxicity or gastrointestinal reaction were similar between the underweight/normal category and the overweight/obese category (all p values were greater than 0.05).

## Discussion

In recent years, an inverse relationship has been found between body weight and breast cancer in most studies. Higher BMI is correlated with worse prognosis in both pre- and postmenopausal women with breast cancer in most, but not all, published studies [14]. However, whether increased BMI has an adverse effect on the efficacy of breast cancer treatment modalities, especially chemotherapy, remains controversial. Neoadjuvant chemotherapy trials may act as a possible approach to address this issue. Currently, the pCR rate is considered to be the most important indicator of the efficacy of neoadjuvant chemotherapy because patients with pCR would achieve nearly perfect long-term survival [15], [16]. Our study has demonstrated that patients with higher BMI were less likely to achieve pCR, which is consistent with the findings from Litton et al. [10]. Because this study is based on a prospective trial with a single NCT regimen, it has offered additional concrete evidence of the relationship between BMI and pathological response to NCT. Furthermore, this is also the first study focus on this issue in Asian populations (Chinese).

Although some data have suggested that higher BMI is associated with a more advanced stage of breast cancer in terms of tumor size [17], lymph node involvement [18] and hormonal status [19], we only found a significantly different distribution of menopausal status among the BMI categories. This finding is partly due to the standardized inclusion criteria of this trial. Moreover, multivariate analysis showed that BMI was an independent predictor of pCR, regardless of other clinical or pathological variables.

Through the subgroup analyses, we found that the effect of BMI on chemotherapy response was not straightforward. It was significantly correlated with pCR in post-menopausal women, but not in pre-menopausal women. This result could possibly be explained because the effect of the continuous production of estrogen by the peripheral adipose tissue of obese women that leads to the indirect advantage of chemotherapy based on the inhibition of ovarian function would be minimized, especially in post-menopausal women [20]. Theoretically, this effect should be more notable in HR positive tumors; however, we found that the predictive value of BMI was significantly shown in HR- patients but not in HR+ patients. Except for the statistical bias induced by the low pCR rate of the HR+ tumors, this finding suggests that there may be other underlying relationships between chemosensitivity and BMI regardless of the ER/PR pathway. In fact, the effect of BMI on breast cancer based on HR status has already been suspected in many previous studies [21], [22], [23].

Although obese women are likely to have increased treatment-related complications, such as radiation complications, lymphedema, and surgical wound [9], [24], there is no current consensus regarding the relationship between obesity and chemotherapy-related complications. However, some clinicians tend to reduce the chemotherapy dose in cases of complications induced by chemotherapy overdoses. In this study, the treatment dose is standardized according to the actual body weight. The incidence of treatment-related adverse events, such as hematologic toxicity, liver toxicity, neurotoxicity and gastrointestinal reactions, were similar in the underweight/normal patients and the overweight/obese patients. Because previous data has indicated that obese patients receiving intravenous CMF have higher leukocyte nadirs than the lean patients [25], it is plausible that obese and overweight patients are possibly under-dosed with currently scheduled doses.

In conclusion, our study has provided additional evidence of the adverse effect of BMI on NCT for breast cancer. Because obesity/overweight is correlated with a lower pCR rate, clinicians should be aware of high BMI as a valuable predictor of poor response to NCT. Attention to a patient’s BMI at diagnosis, especially in post-menopausal patients, may result in more effective NCT and therefore a better outcome [10], although it is not yet possible for us to analyze the relationship between BMI and patients’ prognosis after NCT. Further approaches to investigate the mechanism governing this effect on the treatment modality and to develop a more appropriate schedule for calculating the NCT dose for high-BMI patients should be considered.
